# A Qualitative Study of Barriers and Enablers of Physical Activity among Female Emirati University Students

**DOI:** 10.3390/ijerph18073380

**Published:** 2021-03-24

**Authors:** Nicola W. Burton, Bonnie L. Barber, Asaduzzaman Khan

**Affiliations:** 1 School of Applied Psychology, Griffith University, Brisbane 4122, Australia; 2 Griffith Health Executive, Griffith University, Southport 4215, Australia; b.barber@griffith.edu.au; 3 School of Health and Rehabilitation Science, The University of Queensland, Brisbane 4072, Australia; a.khan2@uq.edu.au

**Keywords:** women, exercise, influences, Arab, college

## Abstract

Interventions to promote physical activity participation should reflect social and culturally relevant influences of the target demographic. The aim of this study was to explore perceptions of barriers to and enablers of physical activity participation among female Emirati university students. Five semi-structured focus groups were conducted (*n* = 25). Participants were asked open-ended questions about benefits, barriers and enablers of physical activity, and recommendations to promote participation. Emergent themes were identified using Nvivo software. Commonly identified benefits included improved health, weight management, improved mood, and stress reduction. The main barriers were low family support, competing time demands from domestic and academic activities, lack of convenient access to women-only facilities, and hot weather. The main enablers and recommendations related to social support from family and friends, accessible and low-cost women-only facilities, and structured supervised sessions. Findings suggest that there are specific social-cultural influences of physical activity among female Emirati university students. Approaches to promote participation could include identifying benefits consistent with family and cultural values, using social media for education, support and modelling, on campus supervised physical activity sessions integrated with the academic timetable, low-cost women-only opportunities in the local residential area, and support for home-based activities.

## 1. Introduction

Physical activity is associated with a reduced risk of all-cause mortality, cardiovascular disease, type-2 diabetes, hypertension, breast cancer, colon cancer, gestational diabetes, ischemic heart disease, and ischemic stroke [1]. Physical activity is also associated with a range of psychological health benefits including reduced risk of depression, anxiety and stress, as well as improved mood [2,3]. For general health benefits, it is recommended by the World Health Organization (WHO) that adults do at least 150 min per week of moderate intensity aerobic physical activity, or at least 75 min of vigorous intensity aerobic physical activity throughout the week, or an equivalent combination of moderate and vigorous intensity activities [4].

Physical activity participation appears to decrease during the transition from adolescence to young adulthood [5,6], which is a time that includes the years spent at university. University students typically report low levels of physical activity, with participation ranging between 30 and 50%, and lower rates among women than men [7,8]. Research with university students in the Gulf Cooperation Council countries identified levels of physical activity ranging from 25 to 47% [9,10,11,12,13,14], which is slightly lower than university students in Western countries [8].

Evidence on the barriers to and enablers of physical activity within specific demographic groups, such as university students, can inform interventions to promote participation. The number of perceived barriers to physical activity significantly increases from high school to university [15] and physically inactive students report a significantly higher number of barriers to physical activity participation than active students [10]. University students typically report lack of time and cost as key barriers [16,17,18]. Lack of social support from parents and/or friends has also frequently been identified [19,20,21]. As a corollary to this, social support has been commonly reported as an enabler of physical activity participation in university students [22,23]. Other commonly identified enablers are availability of lessons and facilities at a reasonable cost [19], on-campus programs [24], and free time [19].

Such evidence from physical activity studies with university students in Western cultures may not be directly applicable to university students in Arab countries due to distinct sociocultural differences, in particular for women. For example, less autonomy with decision making has previously been reported as a key barrier to physical activity among female Kuwaiti university students [25]. A recent review of studies in the Arab region found lower levels of physical activity among women than men in the United Arab Emirates, and cited evidence from a range of studies to attribute this to a convergence of general and gender norms including parents favouring educational and spiritual activities, conservative dress that is unsuitable for physical activity, the need for women to be chaperoned in public spaces, a lack of gender-segregated facilities, a cultural value of comfort and avoiding physical exertion, and the view that public spaces are not appropriate for physical activity [26]. More evidence is needed from culturally and linguistically diverse groups to tailor the development of socially and culturally sensitive interventions. The aim of the current study, therefore, was to examine perceptions of barriers to and enablers of physical activity participation among a group of female Emirati university students.

## 2. Materials and Method

This study was awarded ethical approval by the Human Ethics Research Committee at The University of Queensland (#2017000013).

### 2.1. Participants and Procedure

All participants were recruited from a Higher Education Institute in the Middle East. The university has separate campuses for men and women. Participants were required to be a woman aged over 18 years, and a United Arab Emirates (UAE) national and native Arabic speaker. A separate member of the research team who was known at the Education Institute attended classes at the female student campus, discussed the study with students, and verbally invited participation. Students were given a written information sheet and those interested were asked to complete a written consent form. After providing consent, a date, time and location for the focus group discussions (FGDs) were agreed upon with the participants.

Five FGDs were conducted in a private meeting room on the university campus, and led by the same member of the research team. The FGDs lasted 40–60 min. The FGDs were audiotaped and written notes were taken. All FGDs were conducted in English, which is the teaching language of the university. After the discussion groups, participants completed a short demographic questionnaire online.

### 2.2. Materials

The FGD guide is presented in Table 1. This was developed by the researchers, with questions on benefits of, barriers to, enablers of, and recommendations to improve physical activity participation for female Emirati university students. Participants were initially asked to discuss benefits of physical activity as an ice-breaker activity. Clarification probes were used to explore participants’ responses about intrapersonal (i.e., relating to the individual), interpersonal (i.e., relating to other people) and contextual (i.e., relating to the academic, physical and cultural setting) factors.

The online demographic questionnaire included items about age (years), height and weight (used to derive body mass index (BMI)), general health (excellent, very good, good, fair, poor), ability to manage on available income (easy, not too bad, difficult some of the time, difficult most of the time, difficult all of the time), and life satisfaction (rated from 1–10; 10 being high). Some data were categorized for descriptive purposes, e.g., age was categorised as 18–20, 21–24, 25+ years; life satisfaction was categorised as 1–3 = low, 4–6 = moderate, 7–10 = high.

### 2.3. Data Management and Analysis

Data from the audiotape were transcribed verbatim and entered into Nvivo. The data transcripts were read and analysed by the separate member of the research team. The data were coded and organized into major and minor themes, according to frequency, which were then discussed with the senior author (NWB) to reach consensus. Emergent themes were labelled using constructs from the Theoretical Domains Framework [27], and are presented using the headings: intrapersonal, interpersonal, and contextual (academic, cultural/environmental).

## 3. Results

After the fifth FGD, it was decided that a saturation point had been reached as no new themes had emerged. A total of 25 female university students completed the FGDs. The mean age of participants was 20.4 years (SD 2.6) and 40% were categorised as overweight/obese (BMI > 30). The majority of the participants reported high life satisfaction and excellent/very good health. Additional characteristics of the participants are presented in Table 2.

### 3.1. Benefits of Physical Activity Participation

Major themes identified for benefits of physical activity participation were improved mood, improved health, weight management, disease prevention, and stress reduction. Minor themes identified were disease management, reducing “negative energy” and improving self-confidence, meeting new people, social interactions, improving focus and concentration for academic work, and reducing academic related stress.

### 3.2. Barriers to Physical Activity

All the identified barrier themes, with example participant statements, are summarised in Table 3. Strong interpersonal and contextual themes emerged. Commonly reported factors were low support from family, and competing time demands from household/family responsibilities. Contextual barriers included lack of time because of a busy academic schedule, lack of suitable accessible facilities and hot weather.

At the intrapersonal level, participants described that some women preferred more passive leisure activities, in particular using social media.

“A lot of them would just sit and use their laptops instead of going outside and doing exercises”

At the interpersonal level, low support from family members was one of the most commonly cited barriers to physical activity. It was said that parents and other family members may not encourage women to do physical activity if physical activity was perceived as primarily for weight loss.

“Families think differently to us and maybe sometimes when you are slim they ask why are you going to exercise—they don’t want you to be more slim so they prevent you from doing anything. Like my friend is not allowed to do exercise because she is slim—her family will not allow her”.

Another reason for low family support was related to sociocultural norms regarding appropriate lifestyle activities for women. Participants described needing family permission to engage in activities, or support to enable participation (e.g., transport, money).

“Some people have a culture where the family don’t let them go to gyms and do exercise. Like in UAE, the family - we are more private in our life and they don’t like the girls to go out and do exercise. Parents don’t like it”.

“Doing exercise disagrees with the Emirati culture—the parents are not used to letting the girls go out at 6pm and 8pm to do walking. And there is no time in the day to do exercise”.

“Some parents think that you are a girl and you should learn the kitchen work and you don’t do exercise—this is from the culture”.

Many women described that family and domestic duties created a competing time demand against physical activity. The women would attend university in the day and then return home to care for the household, which resulted in little discretionary time.

“Because most Emirati women are working mothers they don’t have time from their work and they have to take responsibility for their children so they don’t have time for exercise”.

“Not enough time, home responsibilities—taking care of the children, responsibility for other family like our mothers”.

At the contextual level, many of the students described that the intense academic study and assessment schedule left little time for physical activities. Additionally, it was noted that many of the physical activities held at the campus sports complex were at times that clashed with classes, and therefore prevented participation.

“We have many college work and exams and projects, and we don’t have time for gym and exercise”.

“The schedule is really busy for us and it’s not convenient with the schedule of the sports complex. We can’t manage it with our studies”.

Lack of suitable, accessible and affordable physical activity facilities was commonly identified as a barrier. Because of sociocultural reasons, women-only physical activity facilities were required, but it was reported that many of these were expensive to join and located far away from the home.

“And as we are women, we don’t have places to do exercises. Maybe two or three places we have—we don’t have enough clubs for ladies only, and even if there is, it is expensive. Before was cheaper but now double the price. We have ladies’ clubs but it’s expensive”.

“It is not only the gym that we want to go to—there are also parks and there are some for ladies but there are not enough of them and they are a long distance away. Maybe I live in Sharjah but the place is in Abu Dhabi and I have no way to get there”.

The climate of the UAE was also identified as a significant barrier to physical activity. Many of the women said they would like to do outdoor activities, but the hot weather made this uncomfortable. Some women wore traditional clothing in public, and this heightened the discomfort for specific activities such as walking.

“Because it is too hot, we cannot go outside and do exercise especially because we are wearing abaya and shayla, it is very hot for all weather. It is a very big problem to us because sometimes when we go to a walk they tell us that you have to wear the sport dress and come and walk so we can’t walk in these areas as we have to wear our abaya and shayla”

### 3.3. Enablers of Physical Activity

All enablers themes are presented in Table 4. Strong interpersonal, contextual and academic themes emerged, which often mirrored the barriers reported previously. Commonly reported enablers were low-cost activities, accessible women-only facilities, friend and family support (including via social media) and physical activity classes integrated with the academic schedule.

At the interpersonal level, social support was commonly reported as a key enabler of physical activity. Social support sources included friends, family, and from social media (e.g., Instagram influencers, etc.). Friends were reported as a preferred source of support, primarily for providing motivation.

“First of all, I think its support from friends—if we go to the sport together and encourage each other to exercise it will help for me especially”.

Family was also an important source of emotional and material support, including encouragement, transport, and costs for membership fees.

“Encouragement from parents is important when the parents give their children help to be more healthy and do activities and sometimes it’s about cost—the parents can give them money to join centres”.

Social media was identified as a source of knowledge and modelling.

“Social media maybe—for awareness and when you see some videos that advise you how to do the activities and what’s the benefits for it. Some applications like Instagram when you see the posts—there are some people who post about the health and the fitness. They put daily tips about the fitness and how you will be a fit person”.

At the contextual level, exercise classes as part of the college curriculum was identified as enabling students to engage in physical activity.

“Before last year there was one subject where the girls go to the gym but now they have cancelled. If this came back it would be good for the girls to take this subject and go to the gym because some girls do not have enough time but if it is in the schedule then it is easier”.

Also at the contextual level, one of the most commonly cited enablers was women-only facilities which were close to the residential area (i.e., within walking distance) and low cost.

“Now they say in each urban area there must be a club close. Now in our area they do it for each area for ladies only. From government, they approve it and each area must have a closed club to go and do exercise and also have activities”.

### 3.4. Recommendations to Improve Physical Activity Participation

Participants identified the importance of engaging with female students to understand their physical activity interests.

“Ask the females what they prefer to do and ask their opinions on what they like”

Suggested types of physical activity for Emirati female university students included jogging, cardio/aerobic classes, cycling, swimming, and Zumba/dance classes. Participants described that activities should be fun and led by an instructor, who should have both fun, motivational, and educational qualities.

“The program should be fun—not only this is activity and do it”

“I think the people (the instructors) have to show that they care about us—they are friendly and funny and confident and motivate us. And tell us the benefit that we can get from each exercise”.

Structured activities were also important, with participants interested in physical activities that were well planned.

“Not just take the ball and go play basketball—it needs more structure—not just trying to show that we are doing sport”.

The majority of participants preferred that physical activities be held at the university sports complex or at classrooms on the campus, and many agreed that this would encourage them to participate during breaks in their academic schedule.

“On the campus—not outside—like they can allocate some classrooms (for activities) on the campus and we can start. If it is closer to the campus then we can come in the break in our schedule”.

## 4. Discussion

This study offers insight into the barriers and enablers of physical activity among female Emirati university students. Commonly cited barriers reflected sociocultural norms for women and included low family support, competing time demands due to domestic responsibilities, and lack of women-only facilities near to home. Other common barriers were competing time demands from academic schedules and discomfort associated with the hot weather. Commonly cited enablers were social support from friends and family, the availability of low-cost women-only facilities and opportunities in close proximity to home, and organised physical activity sessions integrated with the academic schedule. Participants described a preference for activities that were fun, structured, led by a coach, and held on campus. Jogging and cardio/aerobics were commonly cited as preferred types of activity.

Distinct sociocultural factors were described as influencing physical activity participation. In particular, lack of convenient access to women-only facilities was constantly highlighted. This is consistent with a previous review which indicated the paucity of gender-segregated fitness facilities contributed to low levels of activity among women in Arab countries [26]. Accordingly, having women-only physical activity facilities and clubs in local residential areas was identified as enabling participation. The cost of these facilities is important, with comments that clubs need to be more affordable. However, providing free or subsidized women-only facilities in each residential area would be costly. It may be beneficial to conduct environmental analyses to identify underserved areas that could benefit from cost-reduced physical activity facilities—past research has shown that provision of free access to leisure facilities can increase participation in underrepresented groups [28,29]. There could also be opportunities to provide facilities and activity sessions in group residential buildings. This is important as other participants, noted that they were able to do physical activity within, but not outside, the home. This is consistent with previous research with Qatari women who commented on restrictions in participating in activities outside of the home [30]. Home-based resources, such as instructional materials, equipment and digital applications, may enable physical activity participation for these young women.

Some participants indicated that within their culture, there was a lack of awareness of the range of benefits of physical activity participation for women, other than weight loss. Weight loss as a motivation for exercise is more commonly identified among women than men [31]. However, weight loss may not be salient to all women, and an understanding of the broader range of benefits may motivate participation. The main benefits of physical activity identified by the young women in this study were improved mood, improved health, disease prevention, and stress reduction. Other benefits were improving self-confidence, meeting new people, social interactions, improving focus and concentration for academic work, and reducing academic-related stress. More research is needed to identify what physical activity benefits align with cultural and family values, so that participation is seen as advantageous for those young women for whom weight loss is not a concern. Previous research has demonstrated that health literacy is a consistent predictor of physical activity participation [32], including among female university students in the United Arab Emirates [33], and it may also impact on family support for physical activity.

As in previous studies with university students [16,17,18], lack of time was a commonly identified physical activity barrier. Competing time demands included academic work and family responsibilities, as well as social media use. Academic and family commitments have been commonly identified in previous research [10,19], and our study participants suggested on-campus activities, and physical activity sessions incorporated into the academic schedule, as potential enablers of participation. Time spent using social media may reflect the high availability, accessibility and affordability of smart devices and internet use, as well as the range of popular social media applications, such as Facebook, Instagram and Twitter. Digital media statistics estimate that 99% of the population of the United Arab Emirates are active social media users (UAE Social Media Statistics, 2020). Previous quantitative research with American college students has, however, indicated no association between social media use and physical activity [34]. It may be, therefore, that it is not the time spent on social media, but rather the preference for this as a leisure activity, that constrains physical activity. It is interesting to note that in the current study, use of social media, as a source of education and modelling, was also identified as a potential enabler of physical activity participation. Research on physical activity interventions using social media for these purposes among American university students has shown mixed results. One study reported that a social media intervention did not produce greater awareness of physical activity than an education intervention [35]. Another study found that social comparisons via social media were more effective for increasing physical activity than social support [36]. Other research with women provides some promising results when social media is combined with other intervention components. One study with female college freshmen showed a Facebook social support group improved results from a walking and pedometer self-monitoring intervention [37]. Another study with African American women demonstrated that a Facebook and text message intervention decreased sedentary behaviour and increased self-regulation for activity and light-to-moderate activity [38]. Therefore, more research is needed to understand how social media could be used among Arabic-speaking women to support physical activity participation.

As with other studies [10,39], the hot climate was commonly cited as a barrier to participation, and this was particularly salient for outdoor physical activity such as walking, and for women who wore traditional clothing. The temperature in the UAE can reach upwards of 40 degrees Celsius (104 Fahrenheit) in the summer. Past research with women in Qatari demonstrated a significant decrease in physical activity participation (measured by pedometers) in the summer months [40]. However, some participants in the current study expressed an interest in participating in activities that were outdoors. This interest could be considered during planning activity opportunities, for example, having indoor activities in the hotter months and scheduling outdoor activities for early morning or in cooler months of the year.

Participants reported that physical activities should be fun. Given the stressful nature of university life [41,42], students may prefer physical activities that are not evaluative or results-oriented. This may be particularly salient if there is low confidence/competence for physical activity, which is a key (inverse) predictor of participation among women [43]. Fun activities may also be seen as less effortful, competitive, aggressive, and skills-based—characteristics which are often linked to the traditional male stereotype. Activities perceived as inconsistent with feminine stereotypes can risk negative judgements among young women [44]. The reported preference for scheduled activities may reflect time constraints associated with academic demands and family responsibilities. Participants also preferred instructor-led activities, which was consistent with the preferred types of physical activity identified (e.g., Zumba, cardio).

A limitation of the current study is that participants were recruited through convenience sampling and the summary demographics indicate that a high proportion of the participants had excellent/very good health and high life satisfaction, and 60% were categorised within the healthy weight range. Results may have differed if the sample comprised more women with poor health, low life satisfaction/mood, or high body mass index (BMI), as these concerns are associated with specific barriers to physical activity [45,46,47]. We did not assess the physical activity levels of participants, so we cannot make comments about their experience with physical activity. We used self-reported weight and height data, which is often associated with underestimation of BMI, in particular among those with high body weight [48]; however, BMI was not a focus of this study. Focus groups were conducted in the English language, and even though the students attended an English-speaking university, this may have led to constrained communication as English was not the native language of the participants. Focus groups were led by a male researcher, which may have constrained the female participants’ disclosure of more sensitive information.

The main strength of this study is that is provides a descriptive insight into factors which can constrain or enable physical activity among female university students in an Arab-speaking country. It builds on previous research with university students which has also identified enablers and barriers related to social support, convenient facilities, costs, academic time pressures, competing time demands from family/domestic responsibilities, and hot weather. Our research contextualises these factors for this specific demographic group, and highlights the importance of sociocultural processes. This evidence can be used to generate hypotheses about behaviour and inform other studies. One imperative for future research is assessment with families to understand how physical activity among young adult women can be valued in the culture. A recent review of published physical activity interventions in the Arabic-speaking region concluded that culture is critical to success [49], and our research suggests some key sociocultural components such as aligning physical activity benefits and participation with cultural norms and values; use of social media for education and modelling; providing support for home-based exercise; and creating local, affordable, gender-segregated opportunities for physical activity.

## 5. Conclusions

The findings of this qualitative study suggest that there are specific sociocultural factors associated with physical activity participation among female Emirati university students. It is important to note that the potential impact of such factors may be moderated by the strength of sociocultural norms, which will differ across people. This evidence can be used to understand patterns of behaviour and inform the development of culturally sensitive interventions to promote physical activity participation. Family support for young women to engage in physical activities will be important across a range of intervention strategies. At the university level, integrating instructor-led physical activity classes, which include social and fun aspects, into the academic curriculum could be considered. Upstream approaches could focus on providing low-cost women-only physical activity opportunities in local residential areas and support for home-based activities. If successful, such strategies could make a significant contribution to the physical and mental health of Emirati women.

## Figures and Tables

**Table 1:** Focus group discussion guide.

1. Do you think that there are benefits of participating in physical activity? What are these? 2. What makes it difficult to do physical activity? (Probed for: intrapersonal, interpersonal, social, contextual factors e.g., “How might other people make physical activity difficult”) 3. What helps/would help you to participate in physical activity? (Probed for: intrapersonal, interpersonal, social, contextual factors e.g., “What is it about where physical activity is done that could make it easier to do?”) 4. If we wanted to have a physical activity program for female university students here, what would you suggest? 5. Are there any other points you would like to go back on or talk about?

**Table 2:** Summary characteristics of participants (*n* = 25).

Characteristics	*n*	%
Total	25	100
Age group (years)		
18–20	15	60
21–24	9	36
25+	1	4
Body mass index (kg/m^2^)		
Healthy weight (<25)	15	60
Overweight (25–30)	2	8
Obese (>30)	8	32
General health		
Excellent or very good	15	60
Good	7	28
Fair or poor	3	12
Ability to manage on available income		
It is easy or not too bad	14	56
Difficult some of the time	11	44
Life satisfaction		
High	19	76
Moderate	5	20
Low	1	4

Notes. BMI was based on self-reported height and weight.

**Table 3:** Barriers to physical activity among female Emirati university student participants (*n* = 25).

Major Themes	Minor Themes
Intrapersonal	
Intentions “A lot of them would just sit and use their laptops instead of going outside and doing exercises”	Emotions Positive/negative affect “If you have good mood you will do anything. If you are sad you will not do sports” “Some ladies get bored quickly. If they get bored at the activity today they will not go tomorrow” “Some ladies don’t like to go to the gym—they only think for shopping and going out” Beliefs about Capabilities Self-efficacy “People don’t have enough courage to go and do exercise—they are not confident” Reinforcement Punishment- “If they make any exercise they will get tired and their body will hurt” Knowledge “People don’t know about the benefits of physical activities
Interpersonal	
Social Influences Low social support (family)—“Maybe they don’t have the support from their parents” “Parents don’t like the girls to go out and do exercise” Social norms “Home responsibilities—taking care of the children, and for other family like our mothers”	Social Influences Social pressure (friends) “Sometimes my friends will stop me from doing activities”
Contextual: Academic	
Environmental Context and Resources Environmental stressors “The schedule is really busy for us and it’s not convenient” “We have more exams and a lot of projects in the course—all the time we are busy”	
Contextual: Cultural/Environmental	
Environmental Context and Resources	Social Influences
Material resources “There is no gyms here near us to exercise”	Group norms “In other cultures, they accept this muscle for women. In Emirati female, it is not so acceptable”
“Most of the places are mixed for ladies and men so we can’t go there”	Environmental Context and Resources
Environmental stressors “In the summer we cannot do exercise as it is very hot”	Resources “We are only students—for the majority the cost is the problem”

**Table 4:** Enablers of physical activity among female Emirati university student participants (*n* = 25).

Major Themes	Minor Themes
Intrapersonal	
	Behavioural regulation “The solution is to organise our schedule—it’s just one hour for we will do the activities so we can manage it” Knowledge “The knowledge of the importance of sports”
Interpersonal	
Social Influences Social support (friends) “They need motivation and to go with groups of friends. They will encourage” Social support (family) “The parents can give them money to join centers” Social modelling “I watch videos on health care and that will motivate me”	Social Influences Social support “More coaches or trainers coming to our homes because many parents don’t allow us to go to the gym”
Contextual: Academic	
Environmental Context and Resources Resources “They should make time in our schedule to allow us to do exercise”	Social Influences “Maybe they can do workshops in the school and invite the parents to help the children to do exercise” Reinforcement “The college to give course credit to people who participate in the gym programs”
Contextual: Cultural/Environmental	
Environmental Context and Resources Resources—“I think make the clubs near to the home so that people can walk to them and then transport won’t be a problem” “More private places for girls will help but not just gym—like parks for girls” “Make the sports complex nearer to the college to make the student go” Reinforcement “Making offers will help—if they do many exercise then they have to pay less to use the gym”	Environmental Context and Resources Resources “Having the equipment will help. I have a small gym in my house and that makes me excited to do some exercise” “The gym should provide kindergarten so the lady will feel comfortable”

## Data Availability

Data from the audiotape were transcribed verbatim and entered into Nvivo. The data transcripts were read and analysed by the separate member of the research team. The data were coded and organized into major and minor themes, according to frequency, which were then discussed with the senior author (N.W.B.) to reach consensus.
